# Plasma Gelsolin Levels Decrease in Diabetic State and Increase upon Treatment with F-Actin Depolymerizing Versions of Gelsolin

**DOI:** 10.1155/2014/152075

**Published:** 2014-11-12

**Authors:** Neeraj Khatri, Amin Sagar, Nagesh Peddada, Vikas Choudhary, Bhupinder Singh Chopra, Veena Garg, Renu Garg, Ashish

**Affiliations:** ^1^CSIR-Institute of Microbial Technology, Sector 39-A, Chandigarh 160036, India; ^2^Department of Bioscience and Biotechnology, Banasthali University, Rajasthan 304022, India

## Abstract

The study aims to map plasma gelsolin (pGSN) levels in diabetic humans and mice models of type II diabetes and to evaluate the efficacy of gelsolin therapy in improvement of diabetes in mice. We report that pGSN values decrease by a factor of 0.45 to 0.5 in the blood of type II diabetic humans and mice models. Oral glucose tolerance test in mice models showed that subcutaneous administration of recombinant pGSN and its F-actin depolymerizing competent versions brought down blood sugar levels comparable to Sitagliptin, a drug used to manage hyperglycemic condition. Further, daily dose of pGSN or its truncated versions to diabetic mice for a week kept sugar levels close to normal values. Also, diabetic mice treated with Sitagliptin for 7 days, showed increase in their pGSN values with the decrease in blood glucose as compared to their levels at the start of treatment. Gelsolin helped in improving glycemic control in diabetic mice. We propose that gelsolin level monitoring and replacement of F-actin severing capable gelsolin(s) should be considered in diabetic care.

## 1. Introduction

Plasma gelsolin (pGSN) levels have been reported in a number of disease conditions, and its repletion in case of burn and sepsis models has supported it to be a prognostic marker of wholistic health [1]. In humans, plasma form of gelsolin circulates at a very high concentration (about 200 ± 10 *μ*g/mL) and its function is mainly regulated by free Ca^2+^ ions which in turn are about 1 mM in plasma. Ca^2+^-activated gelsolin rapidly depolymerizes and caps F-actin released in plasma upon cell death/rupture, and the process leads to depletion of pGSN when encountered with a sudden flux of F-actin. Studies have shown that pGSN levels decrease significantly in many pathological conditions in humans and in animal models such as acute liver injury, major trauma, myocardial infarction, sepsis, multiple organ dysfunction syndromes (MODS), lung injury, rheumatoid arthritis, hemodialysis, multiple sclerosis, Alzheimer's disease, tick-borne encephalitis, and Lyme neuroborreliosis [1]. Additionally, some other disease conditions and disorders in which pGSN levels have been reported to decline include malaria [2], allogeneic stem cell transplantation [3], hyperoxia in mice [3], oleic acid induced lung injury [4], cecal ligation/puncture model of sepsis, lipopolysaccharide (LPS, endotoxin) challenge [5, 6], and murine stroke [7]. In short summary, pGSN levels are affected in a range of stress conditions. Realizing that pGSN levels decrease under myriad conditions, we wondered if the levels of this protein alter in another commonly occurring metabolic stress condition known as type II diabetes.

Role of gelsolin has been discussed in a number of hormone-secreting tissues, particularly insulin secreting cell lines from hamster [8]. In 2006, Tomas et al. reported that insulin secretion from pancreatic *β*-cells is enabled by remodeling of actin cytoskeleton, which in turn is closely regulated by intracellular gelsolin [9]. These authors showed that modulation of F-actin state by gelsolin plays a critical role in the glucose-dependent MAPK signal transduction which eventually regulates *β*-cell insulin secretion. Another study confirmed its role in *β*-cell survival, where overexpression of gelsolin in *β*-cells led to significant decrease in the number of terminal deoxynucleotidyl transferase-mediated dUTP nick-end labeling (TUNEL)(+) and active caspase-3(+) cells [10]. Additionally, gelsolin knockdown by RNA interference in B1 cells (subclones derived from parental mouse pancreatic *β*-cell line MIN6) confirmed role of gelsolin in delaying apoptosis in *β*-cells [10]. Since PIP2 regulates functioning of gelsolin, role of PIP2 was examined in pancreatic *β*-cell functioning which showed that presence of excess PIP2 disturbed the F-actin remodeling, possibly* via* PIP2 mediated deactivation of Ca^2+^-activated gelsolin leading to apoptosis of *β*-cells [11]. Further, gelsolin has been reported to directly associate with the N-terminal portion of Syntaxin 4 (Syn4) and regulate insulin granule exocytosis [12]. The work proposed a biphasic model of insulin secretion whereby inactive gelsolin clamps Syn4 from functioning, and Ca^2+^ activation of gelsolin leads to their dissociation in turn enabling Syn4 to facilitate insulin exocytosis. Essentially, these publications indicated some correlation of gelsolin in functioning or survivability of *β*-cells which secrete insulin hormone. Improper management of insulin/sugar ratio is the primary factor in type II diabetes. We examined the pGSN values in type II diabetic humans and in animal models, which brought forth that there is a positive correlation between higher blood glucose levels and reduced pGSN values.

## 2. Materials and Methods

### 2.1. Drugs, Chemicals, and Reagents

Expression and purification of the recombinant proteins, namely, recombinant human gelsolin (rhuGSN) and its truncated versions,* namely*, G1–G3, G2–G6, G4–G6, 28–161, and 56–161, have been described earlier [13]. An image of the SDS-PAGE of the proteins used for the experiments here is presented in Supplementary Figure 1 in Supplementary Material available online at http://dx.doi.org/10.1155/2014/152075 which confirms the levels of purity. The generation of polyclonal anti-human gelsolin antisera in rabbit has also been described earlier [13]. Specificity of the polyclonal IgG1 to rhuGSN and pGSN from human and mice samples was confirmed by western blotting and cross confirmation using monoclonal antibody GS-2C4 (Sigma Aldrich). Confirmation and utilization of these results are published earlier [13]. The regression value of the linear fit of our ELISA based detection method using polyclonal IgG1 and recombinant human pGSN is 0.92 ± 0.07 in the range of 40–400 *μ*g/mL. The values were estimated from three independent experiments, and the linear fit was used to estimate pGSN values and their variations in humans and mice used in this study. Further, sensitivity of our detection method is about 6 ng of rhuGSN. For animal experiments, Streptozotocin (STZ) and Sitagliptin were purchased from Sigma Aldrich and Merck, respectively.

### 2.2. Experimental Subjects

#### 2.2.1. Experimental Mice

Inbred male C57BL/6 mice (3-4 weeks of age), BKS.Cg-Dock^7 m^ +/+ Lepr^db^/J (db/db), diabetic mice (6–8 weeks), and their db/m littermates were procured from the animal facility of the institute. The animals were fed pellet diet and water* ad libitum*. All the protocols on mice were approved from the Institutional Animal Ethics Committee of IMTECH and were performed according to the National Regulatory Guidelines issued by Committee for the Purpose of Control and Supervision of Experiments on Animals (CPCSEA), Ministry of Environment & Forests (Government of India).

#### 2.2.2. Experimental Human Subjects

The study on human subjects was conducted after their informed and written consent according to the Declaration of Helsinki and strictly in accordance with the ethical guidelines of the institute and approved by Institutional BioSafety Committee (IBSC) vide Approval number IBSC/20712/20. Healthy subjects with no history of diabetes were recruited as nondiabetic control, whereas individuals showing consistent higher fasting blood glucose and HbA1c were taken as type II diabetic patients. Subjects suffering from any pathological conditions were excluded from the study. Baseline characteristics of the study participants have been given in Supplementary Table 1.

### 2.3. Establishment of Mice Models of Type II Diabetes

#### 2.3.1. Development of High Fat Diet (HFD) + Streptozotocin (STZ) Induced Diabetes

To generate a nongenetic rodent model of type II diabetes with a combination of insulin resistance and insulin deficiency, C57BL6/J mice were fed an HFD in which 60% of kilocalories was from fat for three weeks and then injected with a single low dose of STZ (intraperitoneal at ~95 mg/kg). This was followed by continued HFD feeding for an additional three weeks. After this time, majority of HFD/STZ-treated mice displayed hyperglycemia, insulin resistance, and glucose intolerance. By about ten weeks of age (and 6 weeks of HFD feeding), animals with similar degrees of hyperglycemia and body weight were randomly divided into various control or protein treatment groups. The normal diet-fed mice were used as nondiabetic controls.

#### 2.3.2. Genetic Mice Model of Type II Diabetes

Additionally, BKS.Cg-Dock^7 m^ +/+ Lepr^db^/J (spontaneous diabetic) mice were used as a genetic mouse model of type II diabetes as they develop diabetes spontaneously. Body weight and feed consumption were significantly higher in experimental diabetic mice (db/db and HFD/STZ C57BL/6) than the control mice. These diabetic mice became obese and thereby showed considerably less locomotor activities as compared to control mice.

Blood samples (200 *μ*L) were collected from tail veins of 6–8-week-old db/db and their nondiabetic littermates, that is, control mice (db/m) as well as 10-week-old HFD/STZ induced diabetic and nondiabetic C57BL/6N mice to estimate blood glucose levels using GLUCOCARD 01-mini instrument (Arkray Piramal Medical Pvt. Ltd.). The instrument was routinely calibrated using standard samples of glucose with predetermined concentration. Parts of the samples were outsourced to a diagnostic lab to estimate HbA1c, while pGSN levels were measured in-house.

### 2.4. Quantification of pGSN Levels in Plasma Samples of Human and Mice Using ELISA

An indirect ELISA was employed to determine the pGSN levels in human and mice samples. In all cases, 100 *μ*L of 1 : 50 diluted plasma with antigen coating buffer (100 mM NaHCO_3_, pH 9.2) was used for overnight coating at 4°C in 96-microwell plate (BD-Falcon). Known amounts of rhuGSN were also coated for standard curve generation. After initial incubation, plates were washed with 1X PBS and wells were blocked with 3% bovine serum albumin in PBS (BSA, Sigma). This step was carried out for 4 hours at room temperature to completely saturate excess binding sites. The plates were then washed with PBS containing 0.1% Tween-20 (PBS-T) followed by overnight incubation at 4°C with rabbit antigelsolin polyclonal IgG antibodies. The wells were again washed as above and incubated with horseradish peroxidase conjugated anti-rabbit IgG (Sigma) for one hour at room temperature. This step was followed by washing steps and subsequently, tetramethylbenzidine (TMB, Thermo Scientific) was added in each well. Reactions were stopped after 10 minutes by adding 2 M H_2_SO_4_. The resulting absorbance in each well was measured at 450 nm with a microplate ELISA reader (Lab Tech). pGSN values in the samples were estimated from the linear fit of absorbance values from samples containing predetermined amounts of rhuGSN.

### 2.5. Blood Glucose Levels in Mice after Repletion with Exogenous Gelsolin (and Its Versions) in Oral Glucose Tolerance Test (OGTT)

For all groups of mice, OGTT was performed with slight modifications in the protocols published earlier [14, 15]. Briefly, mice were fasted over night before blood sample was collected (tail snip) to monitor their fasting glucose level or blood sugar value at *T*_0_ (at zero minutes of experiment). They were then given glucose* via* oral route (2 g per kg of body weight). Subsequently, different groups were given 0.2 mL of PBS (vehicle) or 0.2 mL of PBS containing 8 mg of BSA per mouse or the same amount of rhuGSN or its truncated versions (28–161, G1–G3, 56–161, G2–G6, and G4–G6), or Sitagliptin (10 mg/kg body weight), or 10 mg/kg of Sitagliptin and 8 mg rhuGSN or 10 mg/kg of Sitagliptin and 8 mg of 28–161. Thereafter, blood was collected by tail snipping at *T*_15_, *T*_30_, *T*_60_, and *T*_120_ (15, 30, 60, and 120 minutes) and blood glucose was measured using GLUCOCARD 01-mini.

### 2.6. Week Long Treatment of Diabetic Mice with rhuGSN and Truncated Gelsolin 28–161

To evaluate the effects of sustained treatment with gelsolin, diabetic mice were treated with a daily dose of 8 mg of gelsolin or its truncated yet functional version 28–161 or PBS for 7 days. Three groups were made of four HFD/STZ C57 mice each. After a week of treatment fasting blood glucose levels were monitored in different groups of mice.

### 2.7. Gelsolin Levels in Diabetic Mice following Sitagliptin Treatment

Fasting blood glucose levels and pGSN values were monitored in diabetic mice (C57BL/6 HFD/STZ) at the start of Sitagliptin treatment and after two weeks of daily treatment with either PBS or Sitagliptin (10 mg/kg) as per protocols described above.

### 2.8. Statistical Analysis

The results are expressed as mean ± S.E.M. The statistical significance of the difference between test and control samples was evaluated by unpaired *t*-tests. The differences were considered significant if the probability value was ≤0.05. For the graphs presented in this work, *P* values <0.01, <0.05, and <0.1 compared to control values are shown as ∗∗∗, ∗∗, and ∗, respectively.

## 3. Results

### 3.1. pGSN Levels in Diabetic and Nondiabetic Humans and Mice

**Figure 1:**
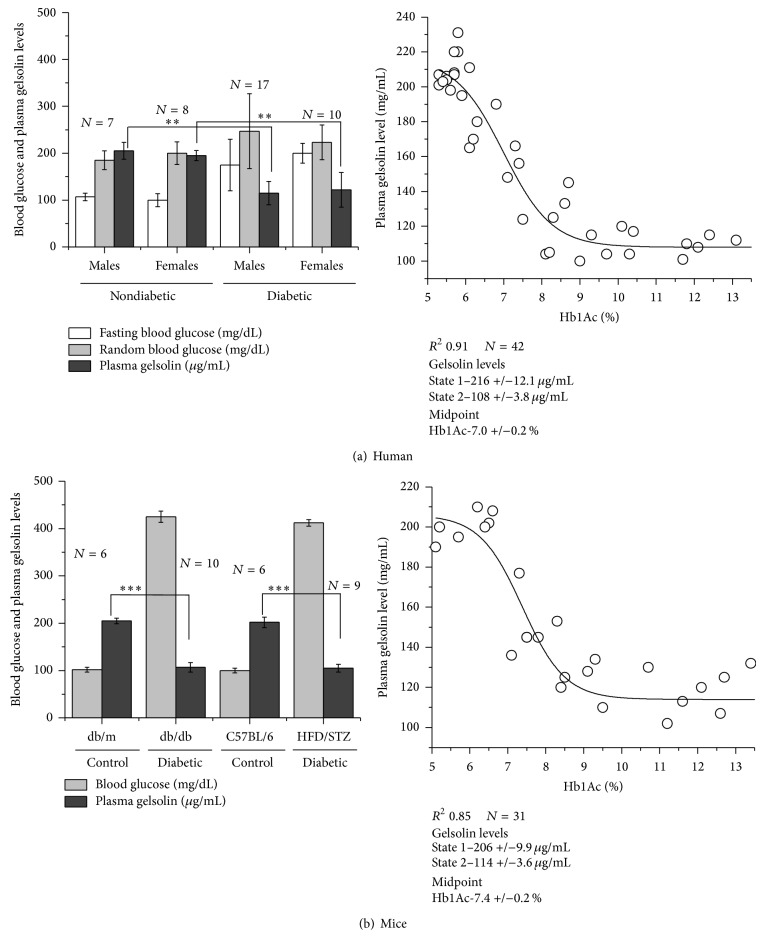
Blood glucose and plasma gelsolin (pGSN) levels in nondiabetic and diabetic human volunteers (a) and mice (b) have been plotted here. The right panels show the correlation between the pGSN values and glycated hemoglobin (HbA1c) percentages in the blood samples. Inset boxes show the parameters obtained upon sigmoidal fit. The results are expressed as mean ± S.E.M. *P* values are as mentioned in Methods (Section 2.8).

Measured pGSN levels in 15 nondiabetic (7 males and 8 females) and 27 diabetic (17 males and 10 females) are plotted in Figure 1(a) (left) along with their fasting and random blood glucose levels. On average, fasting and random blood glucose levels in nondiabetic males and females were about 100 and 200 mg/dL, respectively. In the same sampling, the pGSN values in nondiabetic donors were about 200 ± 8 *μ*g/mL of their plasma. Interestingly, these values were in close agreement with values surveyed earlier [1]. In contrast, diabetic samples with fasting and random blood glucose levels of 195 ± 30 and 230 ± 103 mg/dL, respectively, showed remarkably reduced values (~50%) of pGSN close to 100 ± 25 *μ*g/mL. To obtain a broader view of time, percentage of glycated hemoglobin (HbA1c) was correlated with pGSN values in samples (Figure 1(a), right). The data appeared to change in two states; thus sigmoidal fit was employed to estimate midpoint of transition of pGSN values from healthy to diabetic HbA1c values. Sigmoidal fit to the data showed that while the HbA1c clustered below 6.5% (48 mmol/mol), pGSN values in human subjects were ~216 *μ*g/mL. Data analysis also showed that pGSN values decreased sharply with increase in percentage of HbA1c with midpoint ~7% (53 mmol/mol) which corresponded to ~160 *μ*g/mL of pGSN level (20% below normal). Further, pGSN values decreased to 50% of its value in nondiabetic humans (~108 *μ*g/mL) at HbA1c values higher than 9% (75 mmol/mol). To examine the possibility of interference with the ELISA detection of gelsolin by differential blood glucose levels, we checked the ELISA detection of recombinant gelsolin spiked with glucose but found no difference in the detection compared to the samples with no added sugar (data not shown) thus excluding this possibility.

To see whether pGSN values decrease in mice model of diabetes as well and later use those mice for experimentation, we created two models of type II diabetes. The genetically limited db/db and HFD/STZ models showed substantially higher blood glucose levels than the corresponding nondiabetic db/m and control C57BL/6 mice (Figure 1(b), left). Similar to human samples, nondiabetic mice had pGSN values about 200 *μ*g/mL, while the diabetic mice had a decreased level of pGSN which was about half the value seen in nondiabetic mice, that is, 115 *μ*L/mL. Further, variation in pGSN values in mice as a function of HbA1c values showed that pGSN values remain normal (~206 *μ*g/mL) till HbA1c values are equal or below 7% (53 mmol/mol). pGSN values decreased with increase in HbA1c percentage in mice with midpoint around 7.4% (57 mmol/mol) corresponding to pGSN value of about 156 *μ*g/mL. Our results also indicated that pGSN values sort of settle at a low value of ~114 *μ*g/mL when HbA1c percentage was ~8.7% (72 mmol/mol) or more. These results brought forth two important findings: (1) pGSN values decrease in humans and mice with increase in blood glucose levels and percentage of HbA1c and (2) mice models of diabetes do reflect changes as seen in human patients, and thus GSN repletion experiments in diabetic mice can be correlated with human diabetes care.

### 3.2. OGTT and Repletion of Recombinant GSN and Its Variants

**Figure 2:**
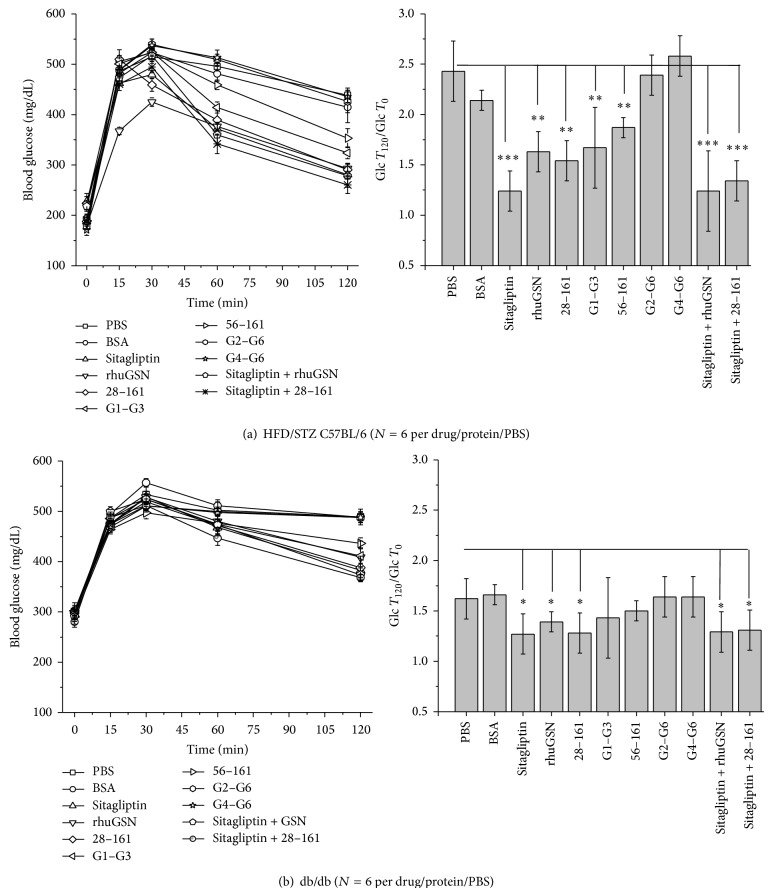
Blood glucose levels measured in oral glucose tolerance test (OGTT) experiments with (a) high fat diet and Streptozotocin induced (HFD/STZ) C57BL/6 and (b) db/db mice are plotted here. Left panels show the variation in the blood glucose value as a function of time, and the right panels have plots of g*T*_120_/g*T*_0_ ratios for different treatments shown in left panels. The results are expressed as mean ± S.E.M. *P* values are as mentioned in Methods (Section 2.8).

OGTT experiments were performed in diabetic HFD/STZ C57 and db/db mice as described in the Methods section. The changes in the measured blood sugar values in both models are plotted in Figure 2, and we have also computed the extent of decrement by mentioning the ratio of blood glucose level at start and end of two hours (g*T*_120_/g*T*_0_). Subcutaneous administration of PBS or albumin in HFD/STZ model showed no decrement in blood glucose levels in these mice (Figure 2(a)). On the other hand, as expected, an oral dose of Sitagliptin brought down blood glucose levels in mice rapidly after 60 minutes. Thus, PBS and BSA showed a value around 2.4 and 2.1, respectively, while Sitagliptin could significantly reduce the g*T*_120_/g*T*_0_ ratio to 1.24. Interestingly, subcutaneous administration of rhuGSN also significantly induced rapid decrement in blood glucose values in mice (g*T*_120_/g*T*_0_ of 1.6). Additionally, the same amounts of different truncated versions of rhuGSN with varying ability to depolymerize F-actin were also injected [13], and we observed that blood glucose levels decreased significantly with administration of 28–161, almost as good as full-length rhuGSN (g*T*_120_/g*T*_0_ ~ 1.54). Similarly, N-terminal half of rhuGSN, that is, G1–G3, could reduce the sugar levels with g*T*_120_/g*T*_0_ ratio of 1.45. Another N-terminal truncated form of rhuGSN, 56–161, with compromised F-actin severing activity could not reduce blood sugar levels in comparison to PBS or BSA. Interestingly, proteins lacking G1 domain and F-actin depolymerizing potential, that is, G2–G6 and G4–G6, showed complete inability to decrease blood glucose levels (g*T*_120_/g*T*_0_ ratios with G2–G6 and G4–G6 were 2.39 and 2.56, resp.). We also tried two combinations, Sitagliptin + rhuGSN and Sitagliptin + 28–161, and found that these two combinations reduced blood glucose level significantly equivalent to that seen for Sitagliptin alone (g*T*_120_/g*T*_0_ ratios for these two combinations to be ~1.24 and 1.34). Overall, there was a significant decrease in blood glucose levels in the mice treated with rhuGSN and 28–161 version of gelsolin as compared to the mice treated with either PBS or BSA (negative control group). However, this effect was more pronounced when gelsolin and 28–161 were administered in combination with Sitagliptin, a standard antidiabetic drug. Moreover, the administration of gelsolin into control animal (nondiabetic) also lowered blood glucose levels (data not shown).

OGTT experiments were repeated in db/db mice (Figure 2(b)). As seen in case of HFD/STZ C57 diabetic mice, application of PBS, BSA, G2–G6, and G4–G6 had no effect in lowering blood glucose levels in genetically diabetic db/db mice. Their g*T*_120_/g*T*_0_ ratios were close to 1.62–1.66. In contrast, application of Sitagliptin and 28–161 achieved a g*T*_120_/g*T*_0_ ratio close to 1.27/8. At same time, single dose of rhuGSN, G1–G3, and 56–161 also brought blood glucose level in db/db mice, and 28–161 showed best potency. As seen in HFD/STZ mice, combined application of Sitagliptin and F-actin severing versions of gelsolin, that is, rhuGSN and 28–161, induced significant decrement in blood glucose levels comparable to either the drug or proteins alone. Even though the effects of pGSN and other F-actin depolymerization competent versions are not as pronounced in db/db model as seen in HF/STZ model, the values were exhibiting the same trend in both the cases. Nonobservance of substantially additive effect suggested that Sitagliptin and F-actin severing competent gelsolins probably function* via* different pathways. These experiments concluded that only F-actin depolymerizing versions of gelsolin can induce rapid decrement in blood glucose levels in OGTT experiments.

### 3.3. Week Long Administration of rhuGSN and 28–161 Keeps Blood Glucose Levels Lower

**Figure 3:**
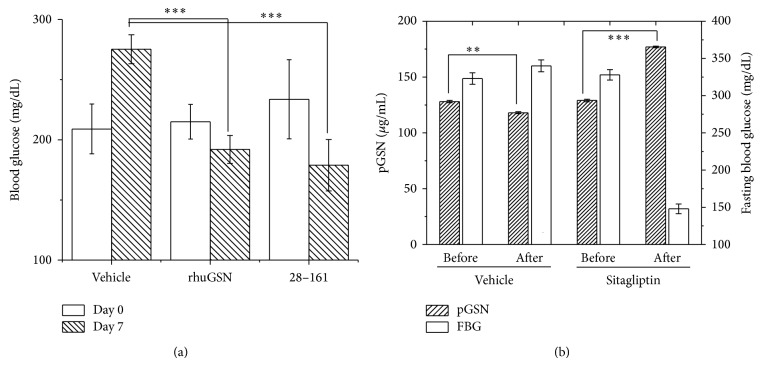
(a) Blood glucose levels on day 0 and day 7 in high fat diet and Streptozotocin induced (HFD/STZ) C57BL/6 diabetic mice treated with daily doses of PBS, rhuGSN, and 28–161 have been plotted here. (b) Variation in measured fasting blood glucose values and pGSN levels in diabetic mice (*N* = 10) before and after daily treatment with antihyperglycemic drug for two weeks has been plotted here. *P* values are as mentioned in Methods (Section 2.8).

Next, we decided to test if per day injection of the two most promising proteins, rhuGSN and its truncated form 28–161, would keep blood glucose level low as done by different antidiabetic drugs. Three groups of mice were injected with 0.2 mL of PBS or 4 mg of rhuGSN and 28–161 in 0.2 mL of PBS every day for 7 days at intervals of 24 hours. Mere injection of PBS did not reduce blood sugar levels in HFD/STZ C57 mice on 7th day, but gelsolin and 28–161 could keep blood glucose levels low (Figure 3(a)). Overall, our results clearly show that a feedback mechanism possibly exists between sugar and pGSN levels in blood.

### 3.4. Treatment with Sitagliptin Increases pGSN Levels in Diabetic Mice

Based on earlier results, we performed another experiment to explore if and how lowering of blood glucose levels in diabetic mice using an antihyperglycemic drug, that is, Sitagliptin, affects pGSN levels. For this, we treated diabetic mice (C57BL/6 HFD/STZ) daily with either PBS or Sitagliptin for two weeks. Their fasting glucose levels and pGSN levels were measured at the onset of experiment and after two weeks of daily treatment with Sitagliptin, and the results are plotted in Figure 3(b). Importantly, we observed that, with decrease in blood glucose levels in diabetic mice following treatment with Sitagliptin, pGSN values significantly increased as seen earlier for nondiabetic control mice (C57BL/6).

## 4. Discussion

Overall, we report for first time that pGSN levels are substantially decreased in diabetic humans and mice. Though these correlate with previous findings that, under myriad stress conditions, pGSN values have been reported to be lower than normal [1], it remains unclear why pGSN values decrease under diabetic conditions. In diabetes, it is well known that body mass reduction occurs due to dehydration, lack of sugar utilization, tissue damage, and protein anabolism [16]. Possibly, the prolonged cell death keeps the spillage of F-actin continuous which leads to depletion of available pGSN from plasma as seen in our experiments. Since pGSN is involved in maintaining healthy condition, diabetics with reduced gelsolin value probably complicate their health complications further. We also found that repletion of gelsolin and its F-actin depolymerizing versions could reduce blood glucose levels. It is important to highlight here that the mice and human GSN are 96% identical, and thus we used rhuGSN and its truncated versions for our repletion experiments in mice. Further, gelsolin levels were found to increase in diabetic animal model upon treatment with antidiabetic drug for 7 days. Since the levels of pGSN after treatment were not above the normal values and the existing literature suggests that biosynthesis of pGSN remains constant here, the increase in pGSN level might be due to less depletion rather than increased biosynthesis of the protein. Earlier, it has been reported that in *β*-cells by depolymerizing F-actin, gelsolin aids in release of insulin [12]. If a similar interaction is possible in plasma as well, then the increased levels of F-actin spilled in plasma may bind insulin and make it nonavailable, thus further complicating the insulin mediated sugar management. Moreover, pGSN is also known to bind to a variety of proinflammatory and bioactive molecules including lysophosphatidic acid, sphingosine 1-phosphate, lipoteichoic acid, and lipopolysaccharide [1]. Upon binding to these molecules, pGSN has been suggested to sequester these bioactive mediators of inflammation and localize inflammatory and immune reactions to the sites of injury. However, since diabetes is primarily a metabolic disorder, the contribution of anti-inflammatory response of pGSN seems less likely to be involved in lowering the blood sugar levels. Further experiments would provide a detailed insight into the mechanism(s) by which the glucose-gelsolin levels are related and whether and how monitoring pGSN values would improve diabetes care.

## 5. Conclusions

This study concluded that pGSN level decreases in type II diabetes in humans and mice. Our results also support that F-actin depolymerizing versions of gelsolin could lower blood glucose levels with gelsolin and truncated form (28–161) performing as good as Sitagliptin. Further, daily dosage of gelsolin(s) exhibited ability to maintain blood glucose levels significantly lower in mice, and treatment with antihyperglycemic drug showed increase in plasma gelsolin levels in mice model.

## Supplementary Material

Supplementary Table summarizes clinical characteristics of human subjects included for type II diabetes studies.
Supplementary Figure shows an image of Coomassie blue stained SDS-PAGE (15%) gel picture showing full length recombinant human gelsolin (rhuGSN) and its truncates along with the molec-ular weight markers is illustrated here.
