# Supplementary material for: Plasma Gelsolin Levels Decrease in Diabetic State and Increase upon Treatment with F-Actin Depolymerizing Versions of Gelsolin
Source: J Diabetes Res. 2014 Nov 12;2014:152075. doi: 10.1155/2014/152075 (PMC4247973; doi:10.1155/2014/152075)
Supplement: Supplementary file 1 — Supplementary Table summarizes clinical characteristics of human subjects included for type II diabetes studies. Supplementary Figure shows an image of Coomassie blue stained SDS-PAGE (15%) gel picture showing full length recombinant human gelsolin (rhuGSN) and its truncates along with the molec-ular weight markers is illustrated here. [file 152075.f1.pdf]

Supplementary Table 1

Clinical characteristics of human subjects included for type II diabetes studies. Values are mean ± S.E.M.

**Parameter**	**Non-diabetic**		**Diabetic**	
Age (Years)	40.6		43.4	
Sex	Male	Female	Male	Female
	N=7	N=8	N=17	N=10
Body Weight (kg)	71.5 ± 2.8	66.3 ± 8.8	73.8 ± 0.6	67.4 ± 2.6
Fasting Blood Glucose (mg/dl)	107.6 ± 4.6	100.5 ± 0.6	175 ± 2.8	204.2 ± 2.2
HbA1c (%)	5.5 ± 1.2	4.9 ± 1.4	8.2 ± 3.8	8.9 ± 3.6
Fasting cholesterol (mg/dl)	157.3 ± 1.4	151.5 ± 6.2	221.6 ± 2.8	227.1 ± 0.9
Fasting triglyceride (mg/dl)	101.1 ± 1.5	111.3 ± 8.9	171.2 ± 2.2	198.5 ± 9.8

Supplementary Fig. 1

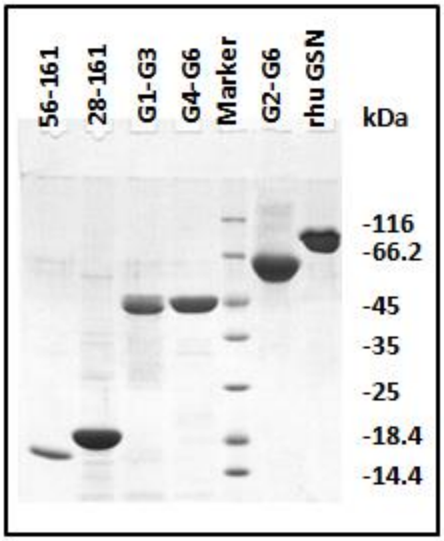

Supplementary Figure 1 A representative Coomassie blue stained SDS-PAGE (15%) gel picture showing full length recombinant human gelsolin (rhuGSN) and its truncates along with the molecular weight markers is illustrated here. Sizes of molecular weight markers are shown on the right as kDa.
